# Impact of Low Hematocrit on On-Pump Coronary Artery Bypass Graft Surgery Outcomes

**DOI:** 10.7759/cureus.91641

**Published:** 2025-09-05

**Authors:** A.M. Nayeem Parvez, Redoy Ranjan, Sheikh Muhammad Bin Faruque, Fatema Farzana, Kaushik Roy, Shanto Barman, Sanjoy Kumar Saha, Sanjay Raha, Md Kamrul Hasan, Asit B Adhikary

**Affiliations:** 1 Cardiovascular Surgery, Impulse Hospital, Dhaka, BGD; 2 Cardiac Surgery, St George's University Hospitals National Health Service (NHS) Foundation Trust, London, GBR; 3 Cardiac Surgery, Bangabandhu Sheikh Mujib Medical University, Dhaka, BGD; 4 Biological Science, Royal Holloway University of London, London, GBR; 5 Cardiac Surgery, National Institute of Cardiovascular Diseases, Dhaka, BGD; 6 Nuclear Medicine, National Institute of Nuclear Medicine and Allied Sciences, Dhaka, BGD; 7 Medicine, Mugda Medical College and Hospital, Dhaka, BGD; 8 Cardiac Anesthesia, Bangladesh Medical University, Dhaka, BGD

**Keywords:** anemia, cardiac surgery, cardiopulmonary bypass, coronary artery bypass grafting, hematocrit

## Abstract

Background: Low hematocrit level is a hematological problem that is frequently encountered during cardiopulmonary bypass (CPB) in patients undergoing on-pump coronary artery bypass grafting (CABG). This study aimed to assess the impact of low hematocrit levels (≤22%) during CPB on the adverse outcomes of on-pump CABG in a large-scale, age- and sex-adjusted Bangladeshi population.

Materials and methods: This comparative cross-sectional study recruited 350 patients, age- and sex-matched, and divided them into two groups: Group A, comprising 175 patients with a hematocrit level of ≤22% on CPB, and Group B, comprising 175 patients with a hematocrit level of >22% on CPB. Univariate analysis curtailed the risk patterns, whereas multivariate logistic regression (LR) analysis observed independent predictors of postoperative adverse outcomes.

Results: The mean age was similar between Group A and Group B (57.82 ± 6.32 vs. 56.97 ± 6.49 years, P = 0.57), with a male predominance in both groups (135 (77.1%) vs. 130 (74.3%), χ² = 0.24, P = 0.78). Univariate analysis showed that Group A had longer mechanical ventilation hours (15.09 ± 2.64 vs. 12.57 ± 3.90, P = 0.002), required more blood transfusions (3.67 ± 1.48 vs. 1.94 ± 0.79 units, P = 0.001), and had longer ICU stays (5.05 ± 0.96 vs. 3.45 ± 1.12 days, P = 0.001) compared with Group B. Postoperative complications were also more frequent in Group A, including acute kidney injury (AKI) (34.3% vs. 14.3%, χ² = 17.96, P < 0.001), deep sternal infection (8.6% vs. 2.9%, χ² = 4.29, P = 0.03), and in-hospital mortality (5.7% vs. 2.9%, χ² = 1.11, P = 0.02). In age- and sex-adjusted multivariate LR analysis, a hematocrit level ≤22% during CPB was significantly associated with adverse outcomes: higher blood transfusion requirements (odds ratio (OR) = 5.95, 95% CI 1.21-9.83, P = 0.001), longer ICU stays (OR = 3.27, 95% CI 1.06-4.24, P = 0.02), AKI (OR = 3.13, 95% CI 1.19-8.14, P = 0.01), deep sternal infection (OR = 1.18, 95% CI 1.31-6.24, P = 0.04), and in-hospital mortality (OR = 1.56, 95% CI 1.17-7.28, P = 0.03). A receiver operating characteristic (ROC) curve analysis yielded an area under the ROC curve of 0.756 (95% CI 0.65-0.86; P < 0.001) for predicting adverse outcomes, with 80.0% sensitivity and 45.0% specificity at a 22% hematocrit threshold.

Conclusions: Hematocrit levels of 22% or lower during CPB are associated with increased morbidity and mortality following on-pump CABG. Maintaining hematocrit levels above 22% during CPB is therefore recommended to improve postoperative outcomes.

## Introduction

Hematocrit represents the proportion of red blood cells in the total blood volume (comprising both red blood cells and plasma), with typical ranges of ~50% in men and 45% in women [1]. It can be measured directly via microhematocrit centrifugation or indirectly using automated counters. The indirect method calculates hematocrit by multiplying red blood cell count (in millions/mm³) by mean cell volume (MCV, in femtoliters), which may introduce variability due to challenges in accurately measuring MCV [1,2]. Over half a million patients undergo cardiac surgery with cardiopulmonary bypass (CPB) each year, and more than half require homologous blood transfusions. Anemia, caused mainly by hemodilution from CPB priming, intravenous fluids, surgical bleeding, and coagulopathy, is a significant concern. While blood conservation programs aim to improve outcomes and reduce costs by tolerating lower hematocrit levels, the optimal safe lower limit for hematocrit during CPB remains undefined, especially in patients with cardiovascular disease, where inadequate perfusion may lead to organ injury [2,3]. Hemodilutional anemia is a commonly employed strategy during CPB, aimed at reducing blood viscosity to maintain normal blood flow under hypothermic conditions without increasing arterial pressure. This approach is thought to reduce complications linked to hypertension, such as aortic dissection and collateral blood flow to the coronary arteries during aortic cross-clamping.

Additionally, using crystalloid priming to induce hemodilution minimizes the need for intraoperative autologous blood transfusion. Although the technique offers several advantages, concerns remain regarding the optimal hematocrit threshold and potential risks associated with acute anemia [3]. Multiple studies have linked low hematocrit levels and red blood cell transfusions to poorer clinical outcomes [2-4].

Fang et al. [4] found that, even after adjusting for patient- and disease-related factors, a hematocrit value of ≤14% in low-risk patients and ≤17% in high-risk patients independently increased mortality risk following coronary artery bypass grafting (CABG) surgery. After adjusting for preoperative variations in patient and disease characteristics, lower hematocrit levels during CPB were significantly associated with higher risks of in-hospital mortality, acute kidney injury (AKI), atrial fibrillation (AF), and stroke. Additionally, lower hematocrit correlated with longer postoperative ventilation time, extended ICU and hospital stays, increased wound infections, and more significant blood loss [3-5].

There are seldom studies in Bangladesh that have investigated the impact of hematocrit levels of ≤22% during CPB on postoperative outcomes in large, age- and sex-adjusted on-pump CABG populations among Bangladeshis.

## Materials and methods

In this study, we performed a comparative analysis of ischemic heart disease patients undergoing on-pump CABG at the National Institute of Cardiovascular Disease, Bangabandhu Sheikh Mujib Medical University, Impulse Hospital and Research Center, and Al-Helal Specialized Hospital in Dhaka, Bangladesh, between January 2018 and June 2025. The study patients were divided into two groups based on the hematocrit level on CPB: 350 patients were enrolled in this study and divided into two groups: Group A included patients with a low (≤22%) hematocrit level during CPB (n = 175), while Group B comprised those with a hematocrit level >22% (n = 175). Patients scheduled for conventional on-pump CABG who met the inclusion criteria were selected through purposive sampling, and informed consent was obtained. This study obtained ethical clearance from the Institute Review Board of Bangabandhu Sheikh Mujib Medical University, with reference number BSMMU/2024/5390. The study was conducted in accordance with the Declaration of Helsinki, and data were encrypted to maintain confidentiality.

Study procedure

Comprehensive preoperative assessments were documented, including medical history, physical examination, and relevant investigations. Antiplatelet therapy with aspirin and clopidogrel was discontinued three and five days before surgery, respectively. Premedication included oral midazolam (7.5 mg) the night before and intramuscular pethidine (75 mg) on the morning of surgery. Patients received oxygen via face mask (50% oxygen) in the operating room, followed by peripheral and internal jugular central venous catheterization under aseptic precautions. A standardized anesthetic protocol was applied to all patients. CABG was performed through median sternotomy. Simultaneously, the great saphenous vein was harvested and prepared, and the internal mammary artery was dissected for either skeletonized or pedicled grafting. After full heparinization, aortic and venous cannulation were performed to establish CPB. Saphenous vein grafts were anastomosed first, followed by left internal mammary artery-to-left anterior descending artery anastomosis. Intraoperative events such as AF, ventricular tachycardia, or ventricular fibrillation were recorded. Hematocrit levels and the number of transfused units were measured intraoperatively.

Fresh whole blood was used to improve hematocrit, enhance oxygen delivery, and provide clotting factors that support hemostasis, thereby reducing risks such as transfusion reactions and electrolyte imbalances. Postoperatively, patients were shifted to the ICU. Initially, they were ventilated with controlled mandatory ventilation followed by synchronized intermittent mandatory ventilation. Clinical monitoring was routinely done using ECG, pulse oximetry, arterial pressure, central venous pressure, and urine output. Extubation was performed when the patient became alert and hemodynamically stable, proper ventilation was maintained, and blood gas levels were within safety limits. Antibiotics and analgesics were administered according to the institution's standard protocol. Postoperative variables like unit of blood transfusion, duration of mechanical ventilation, serum creatinine, postoperative AF, clinically evident stroke, length of ICU stay, postoperative length of hospital stay, blood loss, AKI, and in-hospital mortality were recorded in both groups of patients. AKI was defined, according to the Acute Kidney Injury Network classifications used for diagnosing AKI after cardiac surgery, as a 25% increase in baseline serum creatinine or the need for hemodialysis. The data collection form was filled out and collected throughout the study period, and the results were accumulated at the end.

Statistical analysis

The collected data was entered into a computer, and a data file was created. SPSS Statistics version 28.0 (IBM Corp. Released 2021. IBM SPSS Statistics for Windows, Version 28.0. Armonk, NY: IBM Corp.) was used to evaluate all data. Continuous data was expressed as mean ± standard deviation, and categorical data as a percentage. Differences between the two groups were compared using the chi-square (χ2) test for categorical variables and the Student t-test or, as appropriate, for continuous variables. Results were reported as percentages and odds ratios (ORs) with 95% confidence intervals. Furthermore, Little’s missing completely at random (MCAR) test was conducted to assess the potential impact of missing data on the study's findings. P-values of less than 0.05 were considered statistically significant.

## Results

The study included 350 participants, equally divided between Group A (≤22% hematocrit level on CPB) and Group B (>22% hematocrit on CPB). Baseline demographic and clinical variables were generally well-balanced (Table 1). The overall age of the study population was comparable between the two study groups (57.82 ± 6.32 vs. 56.97 ± 6.49, P = 0.57). The age distribution showed no significant difference (χ² = 2.87, df = 2, P = 0.23), with the majority of participants aged 51-60 years (Group A: 62.9%, Group B: 60.0%). Sex distribution was similar (χ² = 0.24, df = 1, P = 0.78), with males predominating in both groups (77.1% vs. 74.3%). BMI categories were comparable (χ² = 2.11, df = 2, P = 0.34), with overweight patients forming the most significant proportion (62.9% vs. 60.0%). Major comorbidities were also similar between the study groups, including hypertension (65.7% vs. 60.0%, χ² = 0.99, P = 0.80), diabetes mellitus (54.3% vs. 40.0%, χ² = 1.64, P = 0.33), and cerebrovascular disease (5.7% vs. 2.9%, χ² = 1.11, P = 0.55), indicating no significant differences. Furthermore, the left ventricular ejection fraction (P = 0.54) and the number of grafts (P = 0.81) were also comparable among the study groups.

**Table 1:** Baseline characteristics of the study subjects (n = 350) Continuous variables are presented as mean ± SD, and categorical variables are expressed as number (%). Data were analyzed using chi-square test and student’s t-test, as appropriate and the level of significance was set at <0.05. BMI: body mass index, COPD: chronic obstructive pulmonary disease, LVEF: left ventricular ejection fraction, N: number of participants in the study population, SD: standard deviation

Variables		Group A (n = 175) N (%)	Group B (n = 175) N (%)	P-value	Chi-square statistics (χ²)
Age; years (mean ± SD)		57.82 ± 6.32	56.97 ± 6.49	0.57	-
Age group in years	41-50	15 (8.6)	25 (14.3)	0.23	2.87
	51-60	110 (62.9)	105 (60.0)		
	61-70	50 (28.6)	45 (25.7)		
Sex	Male	135 (77.1)	130 (74.3)	0.78	0.24
	Female	40 (22.9)	45 (25.7)		
BMI	Normal (18.5-24.9)	40 (22.9)	35 (20.0)	0.34	2.11
	Overweight (25.0-29.9)	110 (62.9)	105 (60.0)		
	Obese (>30.0)	25 (14.3)	35 (20.0)		
BMI (mean ± SD)		26.87 ± 2.37	27.17 ± 2.39	0.56	-
Hypertension		115 (65.7)	105 (60.0)	0.80	0.99
Diabetes mellitus		95 (54.3)	70 (40.0)	0.33	1.64
COPD		60 (34.3)	40 (22.9)	0.42	1.50
Cerebrovascular disease		10 (5.7)	5 (2.9)	0.55	1.11
LVEF (mean ± SD)		46.51 ± 11.32	45.3 ± 9.23	0.54	-
Number of grafts (mean ± SD)		2.88 ± 0.32	2.85 ± 0.35	0.81	-

To better understand the impact of hematocrit levels during CPB, postoperative outcomes were analyzed between the two groups (Table 2). Notably, these outcomes demonstrated significant differences across several domains, with Group A (≤22% hematocrit level on CPB) experiencing a poorer prognosis than Group B (>22% hematocrit on CPB). Specifically, Group A showed longer mechanical ventilation hours (15.09 ± 2.64 vs. 12.57 ± 3.90, P = 0.002), required more blood transfusions (3.67 ± 1.48 vs. 1.94 ± 0.79, P = 0.001), and had extended ICU stays (5.05 ± 0.96 vs. 3.45 ± 1.12 days, P = 0.001) relative to Group B. In addition, adverse outcomes, such as AKI (34.3% vs. 14.3%, χ² = 17.96, P < 0.001), deep sternal infection (8.6% vs. 2.9%, χ² = 4.29, P = 0.03), and in-hospital mortality (5.7% vs. 2.9%, χ² = 1.11, P = 0.02), were significantly more common in Group A than in Group B, highlighting the association between lower hematocrit levels on CPB and poor postoperative outcomes.

**Table 2:** Distribution of the study subjects by postoperative findings (n = 350) Continuous variables are presented as mean ± SD, and categorical variables are expressed as number (%). Data were analyzed using the chi-square test and Student’s t-test, as appropriate, and the level of significance was set at <0.05. AF: atrial fibrillation, AKI: acute kidney injury, CPB: cardiopulmonary bypass, ICU: intensive care unit, N: number of participants in the study population, SD: standard deviation

Postoperative findings	Group A (n = 175) mean ± SD/N (%)	Group B (n = 175) mean ± SD/N (%)	P-value	Chi-square statistics (χ²)
Mechanical ventilation (hours)	15.09 ± 2.64	12.57 ± 3.9	0.002	
Blood transfusion	3.67 ± 1.48	1.94 ± 0.79	0.001	
Urine volume during CPB	2365 ± 798	2292 ± 925	0.56	
ICU stay	5.05 ± 0.96	3.45 ± 1.12	0.001	
AF	65 (37.1)	45 (25.7)	0.30	2.15
Clinically evident stroke	35 (20.0)	20 (11.4)	0.32	1.98
Adverse outcomes				
AKI	60 (34.3)	25 (14.3)	<0.001	17.96
Pulmonary dysfunction	15 (8.6)	10 (5.7)	0.24	0.68
Deep sternal infection	15 (8.6)	5 (2.9)	0.03	4.29
In-hospital mortality	10 (5.7)	5 (2.9)	0.02	1.11

An age- and sex-adjusted multivariate logistic regression analysis found that a hematocrit level ≤22% during CPB was significantly associated with adverse outcomes: higher blood transfusion requirements (OR = 5.95, 95% CI 1.21-9.83, P = 0.001), longer ICU stays (OR = 3.27, 95% CI 1.06-4.24, P = 0.02), AKI (OR = 3.13, 95% CI 1.19-8.14, P = 0.01), deep sternal wound infection (OR = 1.18, 95% CI 1.31-6.24, P = 0.04), and in-hospital mortality (OR = 1.56, 95% CI 1.17-7.28, P = 0.03) for on-pump CABG patients compared to those with a hematocrit level >22% during CPB (Table 3).

**Table 3:** Age- and gender-adjusted multivariate logistic regression analysis predicting early postoperative complications AKI: acute kidney injury, CI: confidence interval, ICU: intensive care unit, OR: odds ratio

Variables (ref group low (≤22%) hematocrit	OR	P-value	95% CI	
			Lower	Upper
Age	0.56	0.45	0.14	2.56
Sex	0.82	0.78	0.39	1.48
Deep sternal wound	1.18	0.04	1.31	6.24
In-hospital mortality	1.56	0.03	1.17	7.28
AKI	3.13	0.01	1.19	8.14
ICU stays (days)	3.27	0.02	1.06	4.24
Blood transfusion	5.95	0.001	1.21	9.83

**Figure 1:**
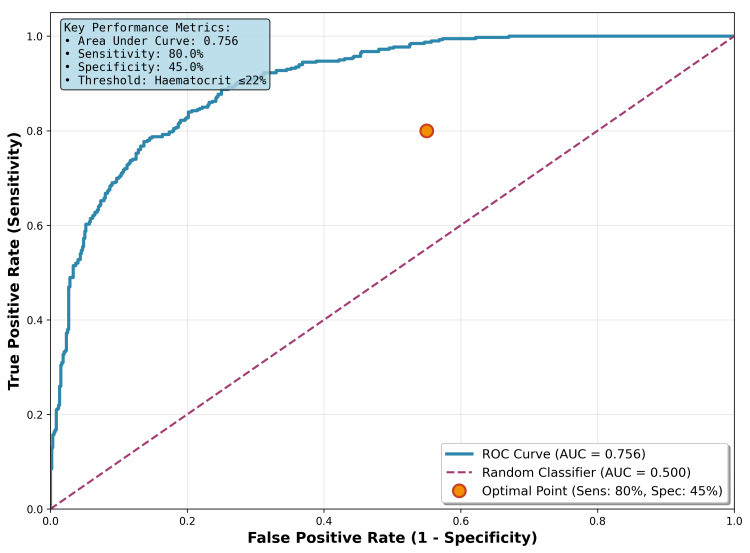
ROC curve assesses the goodness-of-fit of the predictive model for identifying adverse outcomes using hematocrit thresholds of 22.0% AUC: area under the curve, ROC: receiver operating characteristic

Additionally, a receiver operating characteristic (ROC) curve analysis demonstrated an area under the ROC curve of 0.756 (95% CI 0.65-0.86) for predicting adverse outcomes using hematocrit thresholds (Figure 1). At the optimal cut-off point of 22% hematocrit, the model achieved 80% sensitivity and 45% specificity for predicting postoperative complications. Furthermore, Little’s MCAR test was non-significant, suggesting that missing data likely did not affect the study’s findings.

## Discussion

This study reveals that a hematocrit level of ≤22% during CPB in on-pump CABG surgery significantly increases the risks of postoperative morbidities. Patients with low hematocrit face ~2 times the risk of in-hospital mortality, three times the risk of AKI, a 3.3-fold increase in ICU stay duration, and ~6 times the likelihood of needing postoperative blood transfusions. Additionally, low hematocrit is associated with an 18% higher risk of deep sternal infections following on-pump CABG surgery.

The sociodemographic characteristics of our study are consistent with those of different studies across the world, which also report male predominance along with similar characteristics [6-8]. In this study, perioperative data, including graft number and ejection fraction, were comparable between the groups, consistent with other studies worldwide [3,8]. This study revealed significant differences between the two groups in the postoperative period, specifically regarding the duration of mechanical ventilation, ICU stay, and the number of blood product transfusions. Further, the significantly higher in-hospital mortality among the low hematocrit group suggests they receive more blood product transfusions, which aligns with global findings [3,8-10]. A recent study evaluated 6,980 CABG patients; after adjusting for confounders, a lower CPB hematocrit was significantly associated with an increased risk of in-hospital mortality, similar to current study findings [3]. Additionally, in a recent study, Fang and colleagues revealed that low hematocrit levels were associated with more than double the risk of death [4,11,12]. Although a few studies have explored the relationship between the lowest hematocrit during CPB and the risk of post-CABG mortality and adverse outcomes, this is the first study in Bangladesh that found nearly twice the risk of in-hospital mortality.

Like our study findings, several existing studies found that perioperative low hematocrit levels in cardiac surgery have been shown to trigger postoperative complications like acute renal failure [8-10]. Nevertheless, we also found that a low hematocrit is associated with higher blood transfusion requirements and a longer ICU stay, which aligns with findings from several previous studies [11-15]. Patients with lower hematocrit levels showed higher mortality and morbidity rates, which should be factored into their early postoperative management plans, which will help the surgeon and the patient in decision-making and planning the operation.

Study limitations

While the study findings are robust, several limitations must be acknowledged, particularly the small sample size and the short duration of the study, which may affect the robustness of the conclusions. The research was conducted in a multicenter study with a limited follow-up period. The sampling method was purposive, which may have caused outcome bias, and the findings may not be generalizable to broader or more diverse populations. While the study findings are robust, the underlying pathophysiology of low hematocrit levels during surgery and the associated early postoperative morbidity and mortality were not addressed in this study. Additionally, the cross-sectional design precludes the ability to establish causal relationships between variables. Unmeasured or residual confounding factors may also have influenced the results, despite efforts to control for known variables. The lack of long-term follow-up data is also a limitation; therefore, we recommend conducting further studies with larger samples to evaluate the long-term outcomes following on-pump CABG surgery.

## Conclusions

This is the first large-scale, age- and sex-adjusted analysis of a Bangladeshi population undergoing CPB, demonstrating that hematocrit levels ≤22% are associated with a threefold higher risk of AKI and nearly double the risk of in-hospital mortality following on-pump CABG. Our study findings, associated with hematocrit levels of ≤22%, contribute to existing clinical knowledge from a large-scale study in the South Asian population, thereby strengthening the evidence that CPB management guidelines emphasize maintaining hematocrit above 22%, regardless of the temperature protocol. Incorporating hematocrit thresholds into risk-scoring systems could improve the prediction of complications and postoperative hospital stay. Monitoring hematocrit levels during CPB should be prioritized, and proactive strategies aimed at maintaining levels above 22% should be standardized. Risk stratification protocols ought to integrate hematocrit thresholds, and enhanced intraoperative and postoperative monitoring is advisable, particularly for patients presenting with low hematocrit levels during surgery.
